# A graph-based approach for proteoform identification and quantification using top-down homogeneous multiplexed tandem mass spectra

**DOI:** 10.1186/s12859-018-2273-4

**Published:** 2018-08-13

**Authors:** Kaiyuan Zhu, Xiaowen Liu

**Affiliations:** 1 Department of Computer Science, Indiana University Bloomington, 700 N. Woodlawn Avenue, Bloomington, IN, 47408 USA; 2 Department of BioHealth Informatics, Indiana University-Purdue University Indianapolis, 719 Indiana Avenue, Indianapolis, IN, 46202 USA; 3 Center for Computational Biology and Bioinformatics, Indiana University School of Medicine, 410 W. 10th Street, Indianapolis, IN, 46202 USA

**Keywords:** Mass spectrometry, Top-down, Multiplexed mass spectra, Graph algorithms

## Abstract

**Background:**

Top-down homogeneous multiplexed tandem mass (HomMTM) spectra are generated from modified proteoforms of the same protein with different post-translational modification patterns. They are frequently observed in the analysis of ultramodified proteins, some proteoforms of which have similar molecular weights and cannot be well separated by liquid chromatography in mass spectrometry analysis.

**Results:**

We formulate the top-down HomMTM spectral identification problem as the minimum error *k*-splittable flow problem on graphs and propose a graph-based algorithm for the identification and quantification of proteoforms using top-down HomMTM spectra.

**Conclusions:**

Experiments on a top-down mass spectrometry data set of the histone H4 protein showed that the proposed method identified many proteoform pairs that better explain the query spectra than single proteoforms.

**Electronic supplementary material:**

The online version of this article (10.1186/s12859-018-2273-4) contains supplementary material, which is available to authorized users.

## Background

In top-down mass spectrometry (MS), separating similar proteoforms is a challenging problem. A ultramodified protein may have many similar proteoforms with similar weights and different post-translational modification (PTM) patterns. These proteoforms are often not well separated in top-down MS analysis [1]. A *multiplexed tandem mass (MTM) spectrum* is generated when tandem mass spectrometry (MS/MS) is used to analyze two or more proteoforms with similar molecular masses that are not separated by protein separation methods [2]. Despite the complexity of MTM spectra, they have been extensively studied because the interpretation of these spectra provides valuable information about modifications and quantification of proteoforms of ultramodified proteins [1–3]. For example, MTM spectra are frequently observed and analyzed in studies of histone proteins, which play important roles in epigenetics and gene regulation [4, 5].

MTM spectra can be divided into two main types: *heterogeneous* multiplexed tandem mass (HetMTM) spectra and *homogeneous* multiplexed tandem mass (HomMTM) spectra. While HetMTM spectra are generated from proteoforms of two or more different proteins, HomMTM ones from proteoforms of the same protein with different PTM patterns. In data-independent acquisition MS, which has been rapidly developed in the past several years, the precursor ions in a large mass-to-charge ratio (*m*/*z* value) interval are collected for MS/MS analysis, resulting in complex HetMTM spectra [6, 7]. In spectral identification, a HetMTM spectrum is searched against a protein database to find *k* proteins/peptides that best explain the spectrum [2], where *k* is a user-defined parameter. The problem is computational challenging because its search space is proportional to *n*^*k*^, where *n* is the number of proteins/peptides in the database.

In the analysis of HomMTM spectra, we often focus on purified proteins, whose sequences are known. Let *P* be an unmodified protein sequence and *S* a HomMTM spectrum generated from *k* modified proteoforms of *P*. Denote $\mathcal {Q}_{M}$ as the set of modified proteoforms of *P* that match the precursor mass of *S*. The *HomMTM spectral identification problem* is to find *k* proteoforms in $\mathcal {Q}_{M}$ and their relative abundances to maximize the similarity between the theoretical spectra of the proteoforms and the spectrum *S* [1].

DiMaggio et al. first studied the HomMTM spectral identification problem and proposed a mixed integer linear optimization framework for solving it [1]. The proposed framework demonstrated good performance on the analysis of middle-down MTM spectra of histone proteins, but the exponential time complexity of integer linear optimization makes it inefficient for analyzing top-down HomMTM spectra of long protein sequences.

In top-down MS, many software tools have been developed for the identification of proteoforms with PTMs and other alterations [8–12]. However, these software tools are designed for analyzing tandem mass spectra from single proteoforms, not multiplexed ones. Using these tools to analyze an MTM spectrum reports only one proteoform instead of multiple ones.

We formulate the minimum error *k*-splittable flow (ME*k*SF) problem on graphs and convert the HomTMT spectral identification problem to the ME*k*SF problem. To our best knowledge, the ME*k*SF problem has not been studied. However, the maximum *k*-splittable flow (M*k*SF) problem, which is related to the ME*k*SF problem, has been extensively studied and has various applications in commodity transportation and telecommunication network optimization [13–18].

Let *G* be a connected graph with edge capacities, a source vertex, and a sink vertex. A flow is *k*-splittable if it can be decomposed to *k* or less than *k* paths. These paths are neither required to be different, nor edge/vertex disjoint. The M*k*SF problem aims at finding a *k*-splittable flow in *G* from the source to the sink such that the edge capacity constraints are not violated and the flow value is maximized.

Baier et al. [13, 14] first investigated the M*k*SF problem and proved the NP-hardness of the problem on directed graphs for *k*=2. They proposed approximation algorithms with a performance ratio $\frac {2}{3}$ for the maximum 2 and 3-splittable flow problem and presented a $\frac {1}{2}$-approximation algorithm for the general M*k*SF problem. Koch and Spenke [15] studied the complexity and approximability of the M*k*SF problem for different values of *k*≥2 on directed and undirected graphs. In particular, they proved that the problem is NP-hard for *k*=2 on directed and undirected graphs and showed that, for an arbitrary constant *k*, the problem cannot be approximated with a performance ratio better than $\frac {5}{6}$. Koch, Skutella and Spenke [16] decoupled the M*k*SF problem into two steps: the first step called *packing* finds the flow values of the *k* paths in an optimal solution; the second step called *routing* reports the optimal paths of the *k* flow values. The packing procedure was described for general directed graphs, while the routing for graphs with bounded treewidth. Finally, they proposed a polynomial algorithm for the M*k*SF problem on graphs of bounded treewidth when *k* is a constant and presented a polynomial-time approximation scheme when *k* is part of the input.

Unlike the M*k*SF problem, an instance graph of the ME*k*SF problem has capacities on vertices instead of edges. In addition, it is allowed that a flow violates the vertex capacity constrains. That is, the flow value on a vertex may be larger than its capacity. The difference between the flow value and the capacity on a vertex (the flow value may be smaller or larger than the capacity) is defined as the error of the vertex. Let *G* be a connected graph with integer vertex capacities, a source vertex, and a sink vertex. Given a total integer flow value *f*, the objective of the ME*k*SF problem is to find a *k*-splittable flow *F* in *G* from the source to the sink such that the flow value of *F* is *f* and the sum of the errors on the vertices is minimized.

We prove that the ME*k*SF problem is NP-hard when *k* is part of the input and propose a polynomial time algorithm for the problem on layered directed graphs when *k*=2. We tested the algorithm on a top-down MS/MS data set of the human histone H4 protein. Experimental results showed that the proposed method identified many proteoform pairs (path pairs in the graph) that provided better explanation for the query spectra than single proteoforms reported by MS-Align-E [9], an existing tool for the identification of ultramodified proteins.

## Methods

### The ME*k*SF problem

Let *G*=(*V*,*E*) be a directed graph with a source vertex *s* and a sink vertex *t*. Each vertex *v*∈*V* has a positive integer capacity $c(v) \in \mathbb {Z}^{+}$. Let $\mathcal {A}$ denote the set of all simple *s*-*t*-paths (without circles) in *G*. A *k*-splittable *s*-*t*-flow *F* contains *k* pairs (*A*_1_,*f*_1_),⋯,(*A*_*k*_,*f*_*k*_) where *A*_*i*_ is a path in $\mathcal {A}$ and $f_{i} \in \mathbb {Z}^{+}$ is the integer flow value on *A*_*i*_, for 1≤*i*≤*k*. The paths *A*_1_,⋯,*A*_*k*_ may share vertices and/or edges. The flow value of *F* is the sum $\sum ^{k}_{i=1}f_{i}$. Let *F*(*v*) be the set of the pairs (*A*_*i*_,*f*_*i*_) in *F* satisfying that *A*_*i*_ contains vertex *v*∈*V*. The flow value of *v* is the sum of the flow values of the pairs in *F*(*v*), denoted by $f(v) = \sum _{(A_{i},f_{i}) \in F(v)} f_{i}$. The error on *v* is the difference between the flow value and the capacity of the vertex, denoted by *ε*(*v*)=|*f*(*v*)−*c*(*v*)|. The error of *F* is the sum or the errors of all vertices in *G*, denoted by $\varepsilon (F) =\sum _{v \in V} \varepsilon (v)$. The ME*k*SF problem is defined as follows.

#### **Definition 1**

Given a directed graph *G* with integer vertex capacities, a source vertex *s* and a sink vertex *t*, and an integer flow value *f*, the ME*k*SF problem is to find a *k*-splittable flow *F* in *G* from *s* to *t* such that the flow value of *F* is *f* and the error of *F* is minimized.

### The HomMTM spectral identification problem

When a purified protein is analyzed and the target protein is known, the objective of MS analysis is to identify and quantify modified proteoforms of the protein [19, 20]. Although hundreds of PTMs have been found on various proteins, it is common that only several *expected PTMs* are observed on the target protein. For example, expected PTMs on histone proteins include methylation, dimethylation, trimethylation, acetylation, and phosphorylation. In this study, only proteoforms with expected PTMs are considered as candidates in HomMTM spectral identification.

Let $\mathcal {Q}$ be the set of all proteoforms of an unmodified protein *P* with expected PTMs and $\mathcal {Q}_{M}$ a subset of $\mathcal {Q}$ containing all the proteoforms with a molecular mass *M*. For example, when acetylation on lysine residues is the only expected PTM, the set $\mathcal {Q}$ for the protein AKGKL contains three proteoforms AK[acetylation]GKL, AKGK[acetylation]L, and AK[acetylation]GK[acetylation]L. When *M* is the sum of the mass of the protein and the mass shift of an acetylation, $\mathcal {Q}_{M}$ contains only two proteoforms AK[acetylation]GKL and AKGK[acetylation]L. Let *S* be a HomMTM spectrum with a precursor mass *M* generated from proteoforms *Q*_1_,*Q*_2_,…,*Q*_*k*_ of protein *P*. The molecular masses of the *k* proteoforms is the same to the precursor mass of *S*. That is, $Q_{1}, Q_{2},\ldots, Q_{k} \in \mathcal {Q}_{M}$. In practice, errors in the precursor mass of *M* are allowed, and the set $\mathcal {Q}_{M}$ contains all proteoforms whose molecular masses are similar to *M* (the difference is within an error tolerance).

#### **Definition 2**

Given a set $\mathcal {T}$ of expected PTMs, a protein *P*, a HomMTM spectrum *S* with a precursor mass *M*, and a number *k*, the HomMTM spectral identification problem is to find *k* proteoforms $Q_{1}, Q_{2},\ldots, Q_{k} \in \mathcal {Q}_{M}$ and their abundances that best explain the spectrum *S*.

#### Representing the HomMTM spectral identification problem as a graph problem

We will formulate the HomMTM spectral identification problem as the ME*k*SF problem. The proposed method can be applied to tandem mass spectra with various fragmentation methods, such as collision-induced dissociation (CID), higher-energy collision dissociation (HCD), and electron-transfer dissociation (ETD). Here HCD tandem mass spectra are used to explain the method. Only one type of N-terminal fragment ions and one type of C-terminal fragment ions are considered in the method to simply the analysis.

Tandem mass spectra of proteoforms in top-down MS often contain high charge state fragment ions and isotopomer envelopes. The first step in interpreting these spectra is to convert a spectrum into a list of monoisotopic fragment masses using top-down spectral deconvolution tools, such as Thrash [21] and MS-Deconv [22]. In the following analysis, we assume that the spectrum *S* is a deconvoluted tandem mass spectrum.

The target protein *P* is represented as a sequence of amino acids *a*_1_*a*_2_,…,*a*_*n*_. The *i*th prefix residue mass of *P* is the sum of the residue masses of its first *i* amino acids, that is, $p_{i} = \sum _{k=1}^{i} \text {Mass}(a_{k})$, where Mass(*a*_*k*_) is the residue mass of *a*_*k*_. Specifically, *p*_0_=0. The *i*th suffix residue mass of *P* is the sum of the residue masses of its last *i* amino acids, that is, $s_{i} = \sum _{k=n-i+1}^{n} \text {Mass}(a_{k})$. Because of the existence of PTMs, a proteoform *Q* in $\mathcal {Q}_{M}$ may have prefix residue masses different from those of *P*, and the *i*th prefix residue mass of *Q* is the sum of *p*_*i*_ and the mass shifts of the PTMs on the first *i* amino acids in *Q*. Two different proteoforms in $\mathcal {Q}_{M}$ may have the same *i*th prefix residue mass because they have the same mass shifts on the first *i*th amino acids. For example, GK[acetylation]GKL and GKGK[acetylation]L have the same 4th prefix residue mass because the first 4 amino acids in the two proteoforms have the same PTM acetylation on different sites. Similarly, different proteoforms in $\mathcal {Q}_{M}$ may have the same *i*th suffix residue mass. Let $\mathcal {P}_{i}$ ($\mathcal {S}_{i}$), for 0≤*i*≤*n*, be the set of all different *i*th prefix (suffix) residue masses of the proteoforms in $\mathcal {Q}_{M}$. The *i*th prefix residue mass and the *n*−*i*th suffix residue mass of a proteoform in *Q* are called *complementary masses*. Each prefix residue mass in $\mathcal {P}_{i}$ has a corresponding complementary suffix residue mass in $\mathcal {S}_{n-i}$. Let $\mathcal {T}_{i}$ be the set of expected PTMs that can occur on the *i*th amino acid in *P*. A mass *m*_1_ in $\mathcal {P}_{i}$ is a *preceding mass* of another mass *m*_2_ in $\mathcal {P}_{i+1}$ if *m*_2_−*m*_1_ matches Mass(*a*_*i*+1_) or the sum of Mass(*a*_*i*+1_) and the mass shift of a PTM in $\mathcal {T}_{i}$.

**Fig. 1:**
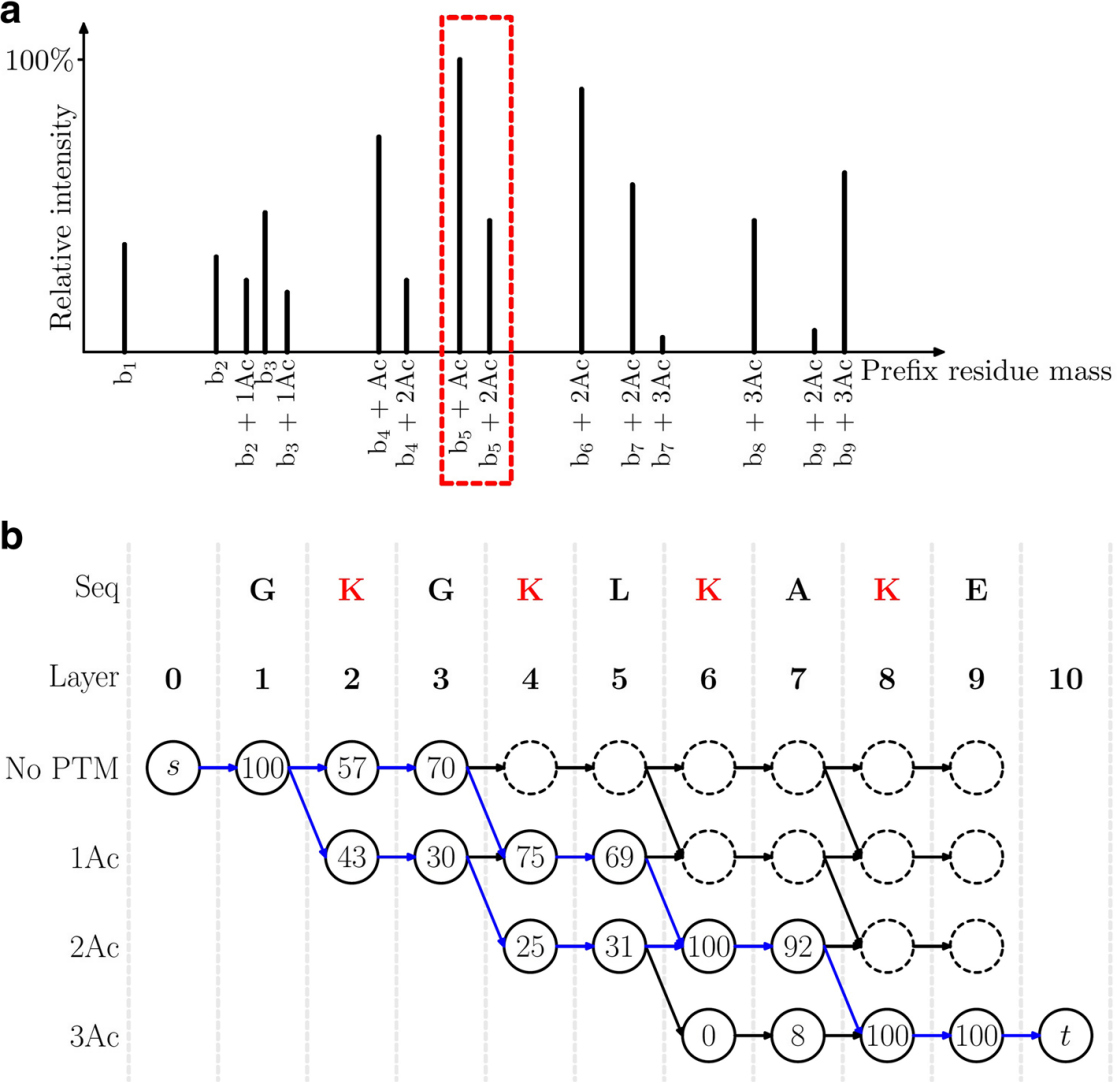
Illustration of the conversion from the HomMTM spectral identification problem to the MS*k*SF problem. A deconvoluted HomMTM spectrum generated from two modified proteoforms of the protein GKGKLKAKE with one expected PTM: acetylation on K, is used as an example. **a** Each peak corresponds to a potential prefix residue mass of a proteoform of GKGKLKAKE satisfying that the prefix residue mass or its complementary suffix residue mass matches an experimental fragment mass. Potential masses for the prefix GKGKL matched to experimental masses are shown in the red dotted box. **b** A graph with 10 layers is constructed based on the masses in $\mathcal {P}_{0}, \mathcal {P}_{1}, \ldots, \mathcal {P}_{10}$ and the peaks in (**a**). Each vertex in layer *i*, 0≤*i*≤10, corresponds to a mass in $\mathcal {P}_{i}$ and those with dotted circles are removed because they are not on any path from the source to the sink. The capacity of a vertex is the ratio (shown in percentage) between the intensity of the mass and the sum of the intensities of all masses corresponding to vertices with solid circles in the same layer. The solution to the MS*k*SF problem is the two blue paths with flows 70 and 30 (in percentage), which correspond to two proteoforms GK[Acetylation]GK[Acetylation]LKAKE with relative abundance 70% and GKGK[Acetylation]LK[Acetylation]AKE with relative abundance 30%

Theoretical masses in $\mathcal {P}_{1},\mathcal {P}_{2},\ldots, \mathcal {P}_{n}$, $\mathcal {S}_{1}, \mathcal {S}_{2}, \ldots, \mathcal {S}_{n}$ are compared with deconvoluted fragment masses in *S* to find matched ones. Mass shifts determined by fragment ion types are added these theoretical masses in the matching because the theoretical prefix or suffix residue masses may have mass shifts compared with their corresponding experimental fragment masses. For example, the mass 18.015 Dalton (Da) of a water molecule is added to theoretical suffix residue masses to match experimental y-ion neutral fragment masses. The raw intensity of a prefix residue mass *m* is the sum of intensities of neutral fragment masses in *S* that match either *m* or the complementary suffix residue mass of *m*, denoted by Inte(*m*). The relative intensity of a prefix residue mass is the ratio between the raw intensity of the mass and the largest raw intensity of all prefix residue masses (Fig. 1a).

A directed graph *G* containing *n*+1 layers is generated from the sets of prefix residue masses $\mathcal {P}_{0}, \mathcal {P}_{1}, \ldots, \mathcal {P}_{n}$ with five steps (Fig. 1b). (1) A vertex is added to the *i*th layer of *G* for each mass in $\mathcal {P}_{i}$. (2) A vertex *u* in the *i*th layer is connected to another vertex *v* in the *i*+1th layer by a directed edge if and only if the mass corresponding to *u* is a preceding mass of the mass corresponding to *v*. (3) The only vertex in layer 0 is labeled as the source vertex, and the only vertex in layer *n* is labeled as the sink vertex. (4) We remove all vertices that are not on any path from the source to the sink. (5) Let *m*_1_,*m*_2_,…,*m*_*k*_ be the prefix masses corresponding to the remaining vertices in a layer of *G*. The capacity of the vertex corresponding to mass *m*_*i*_ is defined as $\frac {\text {Inte}(m_{i})} {\sum _{j=1}^{k} \text {Inte}(m_{j})}$.

Each path from the source to the sink in *G* corresponds to a proteoform in $\mathcal {Q}_{M}$, and the flow of a path corresponds to the relative abundance of the proteoform. Using this method, the HomMTM spectral identification problem is transformed into an ME*k*SF problem on a graph, in which the total flow value is fixed (100 was used in the experiments).

### An algorithm for the ME2SF problem

The ME*k*SF problem is NP-hard on directed acyclic graphs when *k* is part of the input, which can be proved by reducing from the partition problem [23]. (See Additional file 1.) Here we propose a dynamic programming algorithm for the ME*k*SF problem for *k*=2 on layered directed graphs.

A directed graph *G*=(*V*,*E*) is a layered one if there exists a partition of its vertex set *V*={*V*_1_,*V*_2_,⋯,*V*_*h*_}, such that (*u*,*v*)∈*E* if and only if *u*∈*V*_*i*_ and *v*∈*V*_*i*+1_ for 1≤*i*≤*h*−1. Let *G*=({*V*_1_,⋯,*V*_*h*_},*E*) be a layered directed graph in which *V*_1_={*s*} and *V*_*h*_={*t*}. Following the terminology introduced in the studies of the M*k*SF problem, which has many applications on commodity transportation, a flow value pair (*f*_1_,*f*_2_), *f*_1_,*f*_2_≥0, is called a *packing*, and the packing is optimal for the ME2SF problem if there is an optimal ME2SF (*P*_1_,*f*_1_),(*P*_2_,*f*_2_).

Koch et al. has proved that the M*k*SF problem can be solved in polynomial time on graphs with bounded treewidth, including layered directed graphs, when *k* is a constant [16]. The method consists of two steps: the packing step finds candidates for the flow values of the *k* paths in an optimal flow, and the routing step reports *k* paths with the minimum error for each candidate. Similarly, in the proposed algorithm for the ME2SF problem, we first generate candidate packings that contain an optimal one, then find the best routing for each packing.

In the packing step, the total flow value *f* is fixed, a naive approach is to enumerate all possible packings (*f*_1_,*f*_2_) such that *f*_1_+*f*_2_=*f*. The number of candidate packings is *O*(*f*), which may be an exponential function of the length of the input. Below we show that it is sufficient to consider *O*(|*V*|) packings to solve the ME2SF problem.

A set $\mathcal {S}$ of candidate packings with an *O*(|*V*|) size is generated as follows: (1) for each vertex *v*∈*V* with *c*(*v*)<*f*, a candidate packing (*c*(*v*),*f*−*c*(*v*)) is added to $\mathcal {S}$; (2) a special packing (*f*,0) is added to $\mathcal {S}$. The total number of candidate packings in $\mathcal {S}$ is no large than |*V*|+1.

We will prove the candidate set $\mathcal {S}$ contains at least one optimal packing. Let *F*=(*P*_1_,*f*_1_),(*P*_2_,*f*_2_) be an optimal solution to the ME2SF problem, in which *V*_1_ is the set of vertices in *P*_1_, *V*_2_ is the set of vertices in *P*_2_, and *V*_1_≠*V*_2_. Let *v*^∗^ be a vertex in (*V*_1_−*V*_2_)∪(*V*_2_−*V*_1_) with the minimum capacity error. A vertex *v* with *f*(*v*)<*c*(*v*), *f*(*v*)=*c*(*v*), *f*(*v*)>*c*(*v*) is called an *under flow*, *perfect flow*, *over flow* vertex, respectively. The numbers of over flow and under flow vertices in *V*_1_−*V*_2_ are denoted as $n_{1}^{+}$ and $n_{1}^{-}$, respectively; the numbers of over flow and under flow vertices in *V*_2_−*V*_1_ are denoted as $n_{2}^{+}$ and $n_{2}^{-}$, respectively.

#### **Lemma 1**

If *v*^∗^ is not a perfect flow vertex, then $n_{1}^{+} + n_{2}^{-} = n_{1}^{-}+n_{2}^{+}$. That is, the sum of the numbers of over flow vertices in *V*_1_−*V*_2_ and under flow vertices in *V*_2_−*V*_1_ equals the sum of the numbers of under flow vertices in *V*_1_−*V*_2_ and over flow vertices in *V*_2_−*V*_1_.

#### *Proof*

We prove the lemma by contradiction. If $n_{1}^{+} + n_{2}^{-} < n_{1}^{-} + n_{2}^{+}$, then we increase the flow value of *P*_1_ by *δ*=*ε*(*v*^∗^) and decrease the flow value of *P*_2_ by *δ* to obtain a new flow (*P*_1_,*f*_1_+*δ*),(*P*_2_,*f*_2_−*δ*). By increasing the flow value in *P*_1_, the error of each over flow vertex in *V*_1_−*V*_2_ increases by *δ*, and the error of each under flow vertex in *V*_1_−*V*_2_ decreases by *δ* because *δ*=*ε*(*v*^∗^) is the smallest error of the vertices in (*V*_1_−*V*_2_)∪(*V*_2_−*V*_1_). By decreasing the flow value in *P*_2_, the error of each over flow vertex in *V*_2_−*V*_1_ decreases by *δ*, and the error of each under flow vertex in *V*_2_−*V*_1_ increases by *δ*. In addition, the errors of the vertices not in (*V*_1_−*V*_2_)∪(*V*_2_−*V*_1_) do not change. As a result, the error of the new flow is $\varepsilon (F) + \left (n_{1}^{+} + n_{2}^{-} - n_{1}^{-} - n_{2}^{+}\right) \delta $, which is smaller than the error of *F*. This is a contradiction. Similarly, if $n_{1}^{+} + n_{2}^{-} > n_{1}^{-} + n_{2}^{+}$, then the error of the flow (*P*_1_,*f*_1_−*δ*),(*P*_2_,*f*_2_+*δ*) is smaller than the error of *F*, which is a contradiction. □

#### **Theorem 1**

The candidate set *S* contains at least one optimal packing of *G*.

#### *Proof*

Let *F*=(*P*_1_,*f*_1_),(*P*_2_,*f*_2_) be an optimal solution to the ME2SF problem. We consider two cases: (1) *P*_1_ and *P*_2_ are the same and (2) *P*_1_ and *P*_2_ are different. In the first case, the two paths are the same, then (*P*_1_,*f*),(*P*_2_,0) is an optimal solution and $(f,0) \in \mathcal {S}$ is an optimal packing. In the second case, we will prove that there exists an optimal solution *F*^′^=(*P*_1_,*f*1′),(*P*_2_,*f*2′) such that (*f*1′,*f*2′) or (*f*2′,*f*1′) is in $\mathcal {S}$.

Suppose *P*_1_ and *P*_2_ are different and (*V*_1_−*V*_2_)∪(*V*_2_−*V*_1_) is not empty. Let *v*^∗^ be a vertex with the minimum error in (*V*_1_−*V*_2_)∪(*V*_2_−*V*_1_). Without loss of generality, we assume that *v*^∗^∈*V*_1_−*V*_2_. If *v*^∗^ is a perfect flow edge, then *F*^′^=*F* and (*f*_1_,*f*_2_)=(*c*(*v*^∗^),*f*−*c*(*v*^∗^))∈*S*. Otherwise, based on Lemma 1, $n_{1}^{+} + n_{2}^{-} = n_{1}^{-} + n_{2}^{+}$. By changing the flow values (*f*_1_,*f*_2_) to (*c*(*v*^∗^),*f*−*c*(*v*^∗^)), we obtain a new flow *F*^′^=(*P*_1_,*c*(*v*^∗^)),(*P*_2_,*f*−*c*(*v*^∗^)). The difference between the errors of *F* and *F*^′^ is $\left (n_{1}^{+} + n_{2}^{-} - n_{1}^{-} - n_{2}^{+}\right) \varepsilon = 0$. As a result, *F*^′^ is an optimal solution, and $(c(v^{*}), f-c(v^{*})) \in \mathcal {S}$ is an optimal packing. □

In the routing step, we propose a dynamic programming algorithm for finding a flow (*P*_1_,*f*_1_,),(*P*_2_,*f*_2_) for a packing (*f*_1_,*f*_2_) such that the error of the flow is minimized. We first introduce partial flows that are used in the routing algorithm. A path pair with flows (*P*_1_,*f*_1_),(*P*_2_,*f*_2_) is called a partial 2-splittable *s*-*t*-flow if *P*_1_ and *P*_2_ start at the source *s*. The two paths in the partial flow may not end at the sink *t*. The error of a partial flow is defined similarly as the error of a 2-splittable *s*-*t*-flow.

For each ordered vertex pair (*v*_1_,*v*_2_) (*v*_1_ and *v*_2_ may be the same) in a layered directed graph *G*=({*V*_1_,*V*_2_,…,*V*_*h*_},*E*), we define *D*(*v*_1_,*v*_2_) as the minimum error of all partial 2-splittable flows (*P*_1_,*f*_1_),(*P*_2_,*f*_2_) such that *P*_1_ ends at *v*_1_ and *P*_2_ ends at *v*_2_. A vertex pair (*v*1′,*v*2′) ($v^{\prime }_{1}$ and $v^{\prime }_{2}$ may be the same) *precedes* vertex pair (*v*_1_,*v*_2_) if (*v*1′,*v*_1_),(*v*2′,*v*_2_)∈*E*. The error of an ordered vertex pair (*v*_1_,*v*_2_) for a packing (*f*_1_,*f*_2_) is defined as

$$\varepsilon(v_{1}, v_{2}) = \left\{ \begin{array}{ll} |c(v_{1}) - f_{1} - f_{2}| & \text{if}\ v_{1} = v_{2}; \\ |c(v_{1}) - f_{1}| + |c(v_{2}) - f_{2}| & \text{otherwise.} \end{array} \right. $$

Let *T*(*v*_1_,*v*_2_) be the set of all precedent pairs of (*v*_1_,*v*_2_). We use a dynamic programming algorithm to fill out *D*(*v*_1_,*v*_2_) for all vertex pairs *v*_1_,*v*_2_ in the same layer. The recurrence function for computing *D*(*v*_1_,*v*_2_) is

$$D(v_{1}, v_{2}) = \min_{(v'_{1},v'_{2}) \in T(v_{1},v_{2})} D(v'_{1}, v'_{2}) + \varepsilon(v_{1}, v_{2}).$$

After obtaining the value *D*(*t*,*t*) for the sink, we use backtracking to find the best path pair for the ME2SF problem. The time complexity of the routing algorithm is *O*(*l*^4^*h*) where *l* is the largest number of vertices in a layer and *h* is the number of layers in *G*. Since *O*(|*V*|) packings are searched for finding the best solution, the time complexity of the algorithm for the ME2SF problem is *O*(*l*^4^*h*|*V*|). In practice, the value *l* is not large in most cases, and the proposed algorithm is efficient for the ME2SF problem.

## Results

We implemented the dynamic programming algorithm for the ME2SF problem in C++ and tested it on a top-down MS/MS data set of the human histone H4 protein. The experiments were performed on a Linux server with Intel(R) Xeon(R) E5-2680 2.5 GHz CPU.

### Data set

Core histone proteins collected from normal human dermal fibroblasts were separated using a 2-dimensional reverse phase hydrophilic interaction liquid chromatography (RP-HILIC) system. Histone H4 isolated in the first dimension of the separation was analyzed using an LTQ Orbitrap Velos with a resolution of 60k for MS and MS/MS spectra. In total, 1,626 CID and 1,626 ETD spectra were acquired. Details of the experiment can be found in ref. [9].

### Proteoform identification

All top-down tandem mass spectra were deconvoluted using MS-Deconv [22]. In the proposed algorithm, the error tolerances for precursor and fragment masses were set to 15 parts-per million (ppm); the maximum mass difference between the molecular mass of the unmodified protein sequence and the precursor mass of the spectrum was set to 200 Da; five PTMs were treated as expected ones (Table 1). Spectral deconvolution often introduces ±1 Da errors into precursor masses of top-down tandem mass spectra. To address this problem, ±1 Da errors were also allowed in matching precursor masses to the molecular masses of proteoforms.

**Table 1:** Five expected PTMs are allowed in the identification and quantification of histone H4 proteoforms

PTM	Monoisotopic mass (Da)	Amino acids
Acetylation	42.01056	R, K
Methylation	14.01565	R, K
Dimethylation	28.03130	R, K
Trimethylation	42.04695	R
Phosphorylation	79.96633	S, T, Y

With a cutoff of 10 matched fragment ions, the proposed algorithm identified 441 spectra matched to single proteoforms and 184 spectra matched to proteoform pairs. The running time was about 25 minutes and the memory requirement was about 32 GB. If the proteoform pair matched to a spectrum provides explanation for many fragment ions that are not explained by one proteoform, it is highly possible that the spectrum is a HomTMT spectrum. For 39 of the 184 spectra, the proteoforms pairs have at least 10 more explained fragment ions than the single high abundance proteoforms in these pairs. In addition, for 26 of the 184 spectra, the proteoform pairs have at least 20% more explained peak intensity than the single high abundance proteoforms.

### Comparison with MS-Align-E

**Fig. 2:**
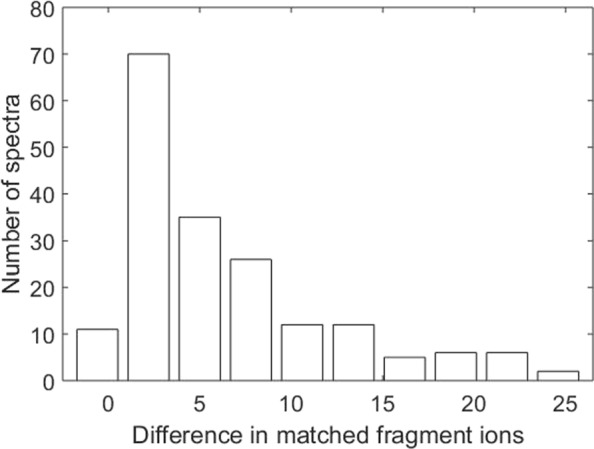
Comparison of the numbers of matched fragment ions. The numbers of matched fragment ions are compared for the 184 spectra identified by both the proposed method and MS-Align-E. For each spectrum, the difference between the number of fragment ions matched to the proteoform pair reported by the proposed method and that matched to the single proteoform reported by MS-Align-E is computed

MS-Align-E [9] was employed to align the histone H4 protein with the deconvoluted tandem mass spectra in the data set. With the same error tolerances and expected PTMs described in the previous subsection, MS-Align-E identified 1037 proteoform-spectrum-matches with at least 10 matched fragment ions. The main reason that MS-Align-E identified more spectra is that unexpected PTMs are allowed in MS-Align-E, but not in the proposed method. The 184 spectra matched to proteoform pairs by the proposed method were all identified by MS-Align-E. For these spectra, we compared the single proteoforms reported by MS-Align-E and the proteoform pairs reported by the proposed method in the number of matched fragment ions and the explained peak intensities. Compared with MS-Align-E, the proposed method increased the number of matched fragment ions by at least 10 for 43 spectra (Fig. 2) and increased the explained peak intensities by at least 20% for 26 spectra, demonstrating that these proteoform pairs better explain the spectra than the single proteoforms.

### Parameter selection

**Fig. 3:**
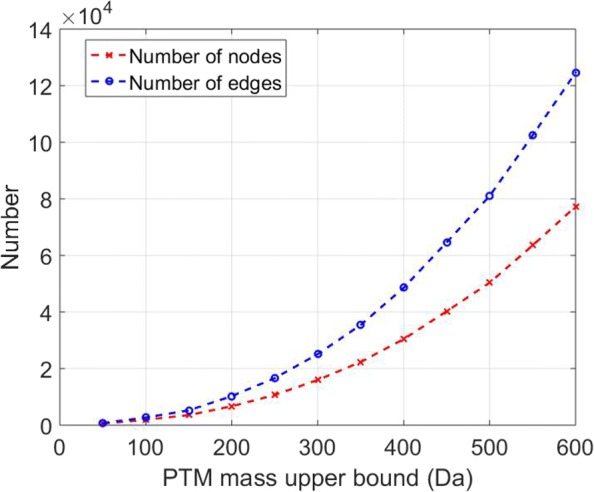
The sizes of graphs used in HomMTM spectral interpretation. The numbers of vertices and edges in the graph generated from the histone H4 protein and five PTMs (acetylation, methylation, dimethylation, trimethylation, phosphorylation) increase significantly when the bound for the sum of mass shifts introduced by PTMs increases from 50 Da to 600 Da

The size of the graph generated from a protein sequence and a set of expected PTMs may be huge due to the combination of PTMs. We can reduce the size of the graph by introducing a bound for the sum of mass shifts introduced by PTMs. When the bound increases from 50 Da to 600 Da, the size of the graph generated from the histone H4 protein and the five expected PTMs increases significantly: the number of vertices increases from 606 to 77,246; the number of edges increases from 761 to 124,633 (Fig. 3). The size of the graph is proportional to *hq*^*t*^, where *h* is the number of layers in the graph, *q* is the largest number of PTM sites in a proteoform, and *t* is the number of expected PTM types. The bound for the sum of mass shifts of PTMs is used to limit the number *q*. In practice, when the number of expected PTM types *t* is 1 or 2, the size of the graph increases slowly with respect to *q* and a large bound 1000 or 1500 Da can be used to allow more PTM sites in a proteoform. When the number *t* is 4 or 5, the size of the graph increases rapidly with respect to *q* and a small bound 500 or 600 Da should be used to guarantee the efficiency of the algorithm.

## Discussion

Because experimental mass spectra contain many noise peaks and miss many fragment peaks, it is not easy to confidently identify more than 2 proteoforms from a HomMTM spectrum. Therefore, we focus on the HomMTM spectral identification problem for *k*=2. The proposed algorithm in the routing step can be extended to the case with *k*>2, but the one in the packing step cannot. A trivial algorithm for the packing step is to enumerate all combinations of flow values for the *k* paths, and the number of candidate packings is *O*(*f*^*k*−1^), where *f* is the total flow value. Coupled with the dynamic programming algorithm for the routing step, the time complexity of the combined method is *O*(*l*^2*k*^*hf*^*k*−1^).

## Conclusions

We formulated the HomMTM spectral identification problem as the ME*k*SF problem on graphs and proposed an efficient algorithm for solving the ME2SF problem on layered directed graphs. The experiments on the histone H4 data set demonstrated that the proposed algorithm is capable of identifying many top-down MTM spectra and gives better explanation for these spectra using proteoform pairs.

## Additional file

Additional file 1 Supplementary material. (PDF 162 kb)

## Abbreviations

CID: Collision-induced dissociation
Da: Dalton
ETD: Electron-transfer dissociation
GB: Gigabyte
HCD: Higher-energy collision dissociation
HetMTM spectrum: Heterogeneous multiplexed tandem mass spectrum
HomMTM spectrum: Homogeneous multiplexed tandem mass spectrum
LTQ: Linear trap quadrupole
ME2SF: Minimum error 2-splittable flow
ME*k*SF: Minimum error *k*-splittable flow
M*k*SF: Maximum *k*-splittable flow
MS: Mass spectrometry
MS/MS: Tandem mass spectrometry
MTM spectrum: Multiplexed tandem mass spectrum
NP-hard: Non-deterministic polynomial-time hard
PTM: Post-translational modification
ppm: Parts-per million
RP-HILIC: Reverse phase hydrophilic interaction liquid chromatography
