# Supplementary material for: A graph-based approach for proteoform identification and quantification using top-down homogeneous multiplexed tandem mass spectra
Source: BMC Bioinformatics. 2018 Aug 13;19(Suppl 9):280. doi: 10.1186/s12859-018-2273-4 (PMC6101081; doi:10.1186/s12859-018-2273-4)
Supplement: Supplementary file 1 — Supplementary material. (PDF 162 kb) [file 12859_2018_2273_MOESM1_ESM.pdf]

A graph-based approach for proteoform identification and quantification using top-down homogeneous multiplexed tandem mass spectra (supplementary material)

Kaiyuan Zhu[1] and Xiaowen Liu[2,3]

[1] Department of Computer Science, Indian University Bloomington

[2] Department of BioHealth Informatics, Indian University-Purdue University Indianapolis

[3] Center for Computational Biology and Bioinformtics, Indiana University School of Medicine

1 Proof of the NP-hardness of the MEkSF problem

In the decision version of MEkSF problem, we are given a graph G with vertex capacities, a flow f, and a number k, the objective is to determine if there are k splittable flow F such that its flow is f and its error is 0.

Theorem 1. *The decision version of the MEkSF problem is NP-complete.*

Proof. We reduce the partition problem to the decision version of the MEkSF problem. Given a multiset S of positive integers, the partition problem is to determine if S can be partitioned into two subsets S_1 and S_2 such that the sum of the numbers in S_1 equals the sum of the numbers in S_2.

For a given instance $S = \{a_1, a_2, \dots, a_n\}$ of the partition problem, we construct an instance of the MEkSF problem. Let $C = \sum_{i=1}^{n} a_i$. The graph contains four layers. The first layer contains only one source vertex s, and the fourth layer contains only one sink vertex t. For each number $a_i \in S$, a vertex $u_{2,i}$ is added to the second layer of the graph and the capacity of $u_{2,i}$ is a_i. Two vertices $u_{3,1}, u_{3,2}$ are added to the third layer and their capacities are $C/2$. Next, we add edges to connect vertices in neighboring layers. For each vertex pair v_1 and v_2 such that v_1 is in layer i and v_2 is in layer $i+1$ (for $1 \leq i \leq 3$), an directed edge is added from v_1 to v_2. The total flow value is set as C and the number k of splittable paths is set as n.

$\rightarrow$ If there is a solution S_1 and S_2 to the instance of the partition problem, we can find an n-splittable flow with error 0 as follows. For each number $a_i \in S_1$, we add the path $s, u_{2,i}, u_{3,1}, t$ to the solution to the MEkSF problem; for each number $a_j \in S_2$, we add the path $s, u_{2,j}, u_{3,2}, t$ to the solution to the MEkSF problem. Finally, the flow that goes through $u_{3,1}$ is $C/2$ and the flow that goes through $u_{3,2}$ is also $C/2$. The total error of the n splittable paths is 0, and the total flow of the paths is C.

$\leftarrow$ If the instance of the MEkSF problem has a solution such that its total flow value is C and its error is 0, then the partition problem has a solution. Let $\mathcal{P} = \{P_1, P_2, \ldots, P_n\}$, a set of n paths from s to t, be the solution to the MEkSF problem. Two observations can be obtained: (1) There are no two paths in $\mathcal{P}$ that go through the same vertex in layer 2. If there exists such a path pair, then at least one vertex in layer 2 does not appear in any path in $\mathcal{P}$ and its flow is 0. As a result, the total error of the n splittable paths is not zero, which is a contradiction. (2) The sum of the flows of the paths that go through $u_{3,1}$ is $C/2$ and the sum of flows of the paths that go through $u_{3,2}$ is also $C/2$. A number $a_i \in S$ is added to S_1 if $\mathcal{P}$ contains a path $s, v_{2,i}, v_{3,1}, t$; S_2, otherwise. Based on observation 1, the assignments result in a partition of S. Based on observation 2, the sum of the numbers in S_1 equals to the sum of the numbers in S_2. $\square$
